# Differentiation of Lipedema Nodules and Angiolipomas Based on the Ultrasound and Histopathologic Findings: A Case Series

**DOI:** 10.7759/cureus.114807

**Published:** 2026-08-19

**Authors:** Deise Vargas, Thais O Foureaux, Ximena Wortsman, Rosa Maria S Sigrist, Rute Lellis, Lucas C Chagas, Paulo Y Saiki, Leila Riedel, Gabriela N Bizareli, Cláudia Fontan, Luciana Zattar, Ana Carina G Silva, Simone A Dini, Juliana Rezende, Daniella Prudente, Eliza P Ducati, Katia Maria D Carvalho, Daniele C Melo, Isabela W Cunha, Walkiria H Bernardi, Alexandre S Bezerra, Alexandre C Amato, Anderson N Bueno

**Affiliations:** 1 Radiodiagnosis, Instituto Magna Opus, São Paulo, BRA; 2 Radiology, TFX Diagnósticos Médicos, Ribeirão Preto, BRA; 3 Dermatology, Universidad de Chile, Santiago, CHL; 4 Radiology, Hospital das Clínicas, Faculdade de Medicina, Universidade de São Paulo, São Paulo, BRA; 5 Pathology and Laboratory Medicine, Rede D'Or, São Paulo, BRA; 6 Radiology, Rede D'Or, São Paulo, BRA; 7 Dermatology, Saiki Dermatology and Trichology, São Paulo, BRA; 8 Medicine, Universidade Federal Fluminense, Rio de Janeiro, BRA; 9 Medicine, Rede D'Or, São Paulo, BRA; 10 Medicine, UltraPlenna, Recife, BRA; 11 Radiology, Hospital Sírio-Libanês, São Paulo, BRA; 12 Medicine, Hospital Universitário Antônio Pedro, Niterói, BRA; 13 Radiology, Foco Ultrassonografia Especializada, São Leopoldo, BRA; 14 Dermatology, LeDerme Skin Lab, Niterói, BRA; 15 Diagnostic Imaging, Instituto de Radiodiagnóstico da Bahia, Salvador, BRA; 16 Diagnostic Imaging, Clínica ED Ultrassonografia Dermatológica, São Paulo, BRA; 17 Diagnostic Imaging, Clínica Bioimagem Diagnósticos, Recife, BRA; 18 Medicine, Faculdade de Ciências Médicas da Santa Casa de São Paulo, São Paulo, BRA; 19 Vascular Surgery, Instituto de Infectologia Emilio Ribas, São Paulo, BRA; 20 Vascular Surgery, Amato - Instituto de Medicina Avançada, São Paulo, BRA; 21 Vascular Surgery, Faculdade de Medicina do ABC, São Caetano do Sul, BRA

**Keywords:** angiolipoma, diagnostic imaging, histology, hyperechoic nodule, lipedema, lipoma, pathology, ultrasound

## Abstract

Hyperechoic subcutaneous nodules in lipedema may mimic angiolipomas but represent an inflammatory and hypoxic-ischemic process rather than a neoplasia, despite tissue expansion. As the painful nodules expand, biopsy is recommended to exclude cancer. In this context, ultrasound (US) has become a pivotal tool for diagnosing and managing these nodules when combined with histopathologic assessment. However, many professionals in the field still have limited knowledge of this topic. In the present case series, the US and histopathologic findings of hyperechoic nodules in two patients with lipedema were compared with those observed in two patients with angiolipoma, with the aim of proposing US criteria to distinguish between these entities and highlighting the importance of accurate differential diagnosis.

## Introduction

Lipedema is characterized by symmetrical accumulation of subcutaneous adipose tissue in the lower and upper limbs, sparing the trunk, hands, and feet [1-3]. It has a progressive and multifactorial nature, affecting approximately 12.3% of the Brazilian female population [4]. Diagnosis is primarily clinical and is based on difficulty in losing weight despite dietary interventions, sensations of heaviness and fatigue in the legs, pain and increased tenderness to palpation, and a tendency toward spontaneous bruising and hematomas [1,4]. Lipedema remains underdiagnosed and is frequently confused with obesity, lymphedema, lipohypertrophy, or multiple lipomatosis [2,3,5,6]. Misdiagnosis may delay appropriate treatment and lead to inadequate approaches, including unnecessary surgical interventions.

Ultrasonography (US) has emerged as an important complementary imaging modality for diagnosing and classifying lipedema, including through the Lipedema Dermal and Hypodermal Classification (LDHC) system [7,8]. Within this system, the presence of painful hyperechoic nodules in the superficial hypodermis is a key finding of LDHC 3 and has been previously described in patients with lipedema [9,10]. Because of their sonographic appearance and subcutaneous location, LDHC 3 hyperechoic nodules may be mistaken for angiolipomas [11].

Histologically, LDHC 3 nodules in lipedema have been associated with hemorrhage, hemosiderin deposition, fibrous proliferation, and focal steatonecrosis within structurally remodeled hypodermal tissue [8-10]. In contrast, angiolipomas are benign tumors composed predominantly of mature adipocytes and a prominent vascular component characterized by an extensive capillary network, which may contain intravascular fibrin microthrombi and be associated with fibrotic tissue and mast cells [11,12].

The lack of established sonographic criteria distinguishing lipedema nodules from angiolipomas may lead to misdiagnosis and potentially result in unnecessary intervention or surgical excision. Defining such criteria is essential to ensure accurate diagnosis and guide appropriate therapeutic management [8-11]. Therefore, this case series aimed to compare the US and histopathologic findings of hyperechoic nodules in patients with lipedema with those of angiolipomas, to propose diagnostic criteria that may support a more accurate differential diagnosis and more appropriate clinical management.

## Case presentation

Four adult patients presenting with painful subcutaneous nodules were included. All patients underwent US examination using high-frequency linear transducers (3-22 MHz) on a Samsung V7 system (Samsung Medison Co., Ltd., Seoul, Republic of Korea, 2024), in accordance with previously established LDHC protocols [7,8]. Sonographic analysis assessed nodule echogenicity, margins, presence of a capsule, Doppler vascularization, and the architecture of the surrounding hypodermis, including the dermal-hypodermal junction (DHJ). Images were acquired and stored in DICOM format.

Biopsies were performed under local anesthesia following US-guided localization and needle marking of the target lesions. Histopathologic evaluation was conducted on hematoxylin-eosin-stained sections by dermatopathologists and included a total of eight nodules: six from patients with lipedema and two angiolipomas.

Case 1

**Figure 1:**
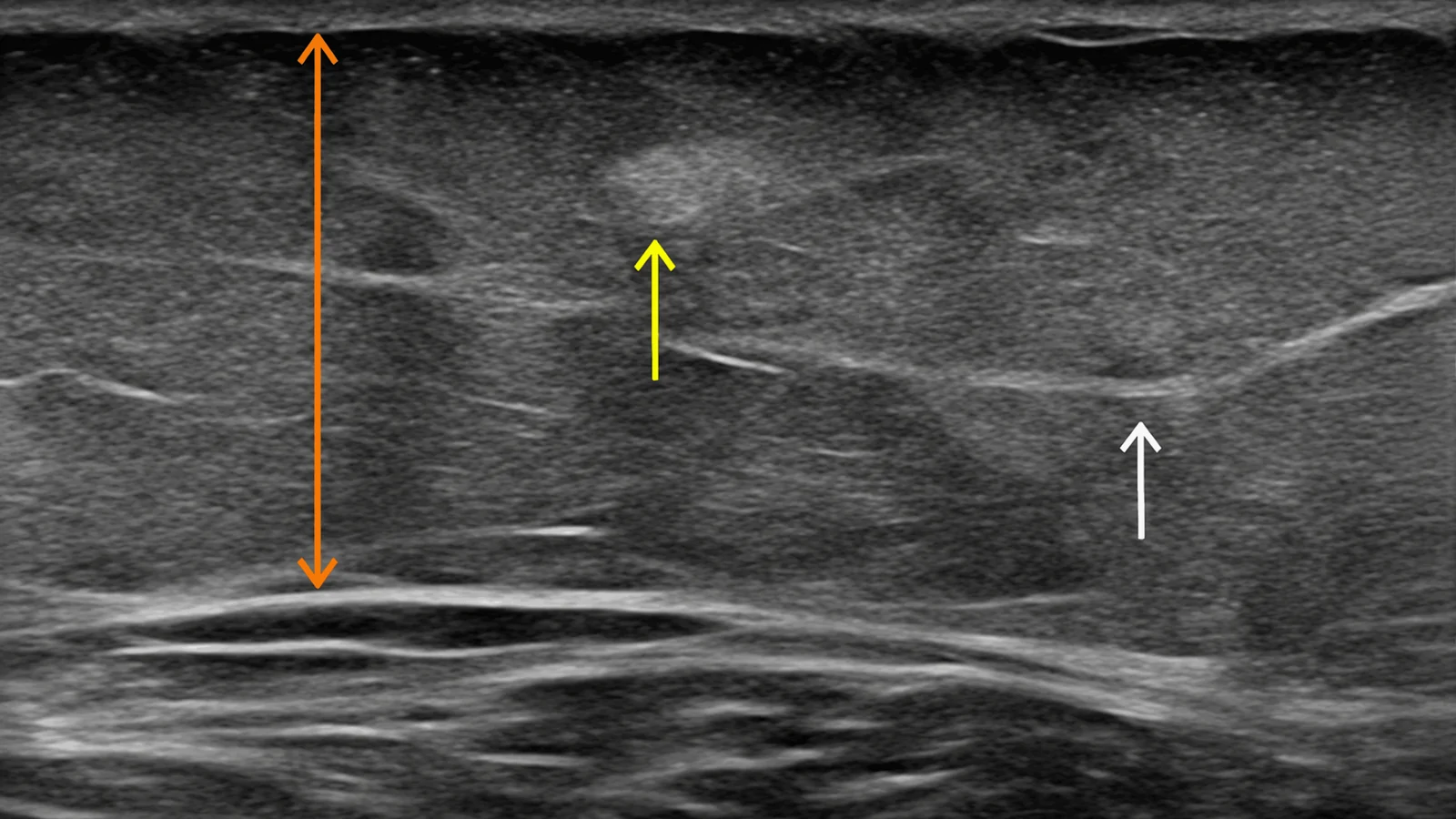
Ultrasound of a thigh nodule in a patient with lipedema Ultrasonographic image of a thigh nodule in a patient with lipedema (Lipedema Dermal and Hypodermal Classification 3) demonstrating thickened hypodermis (orange arrow), bulging deep hypodermis, disorganized superficial hypodermis, irregular septa (white arrow) predominantly in the superficial hypodermis, presence of a hyperechoic nodule (yellow arrow), asymmetric dermal thickening, and an irregular dermal-hypodermal junction.

A 55-year-old female patient had a clinical diagnosis of lipedema involving the thighs, buttocks, and arms, with a 20-year history of lipedema-related symptoms. She presented with one painful LDHC 3 nodule on the posterior aspect of the right arm and another on the posterior aspect of the right thigh as incidental findings. Ultrasound examination of the thigh nodule demonstrated a typical LDHC 3 pattern, including thickened hypodermis, bulging of the deep hypodermis, disorganization of the superficial hypodermis, irregular septa predominantly in the superficial hypodermis, a hyperechoic nodule painful to palpation, asymmetric dermal thickening, and an irregular DHJ. Doppler assessment showed absent or minimal internal vascularity within the hyperechoic nodule (Figure 1).

**Figure 2:**
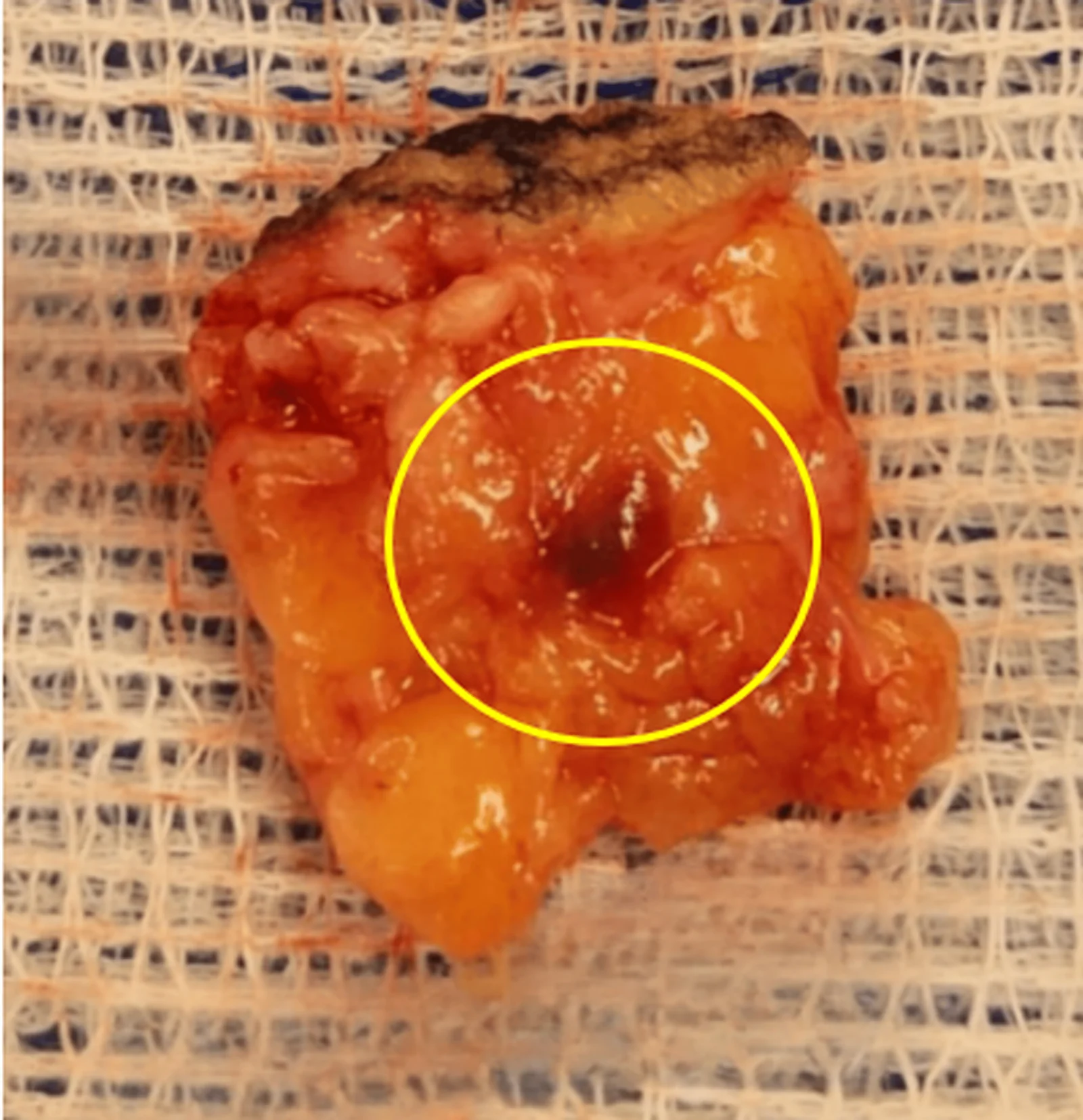
Macroscopic surgical specimen of a lipedema nodule Macroscopic image of the surgical specimen from a thigh nodule in a patient with lipedema (Lipedema Dermal and Hypodermal Classification 3), demonstrating an area of hemorrhage (yellow circle) permeating hypertrophic lobulated subcutaneous tissue.

Macroscopic examination of the excised thigh lesion showed an area of hemorrhage permeating hypertrophic lobulated subcutaneous tissue (Figure 2).

**Figure 3:**
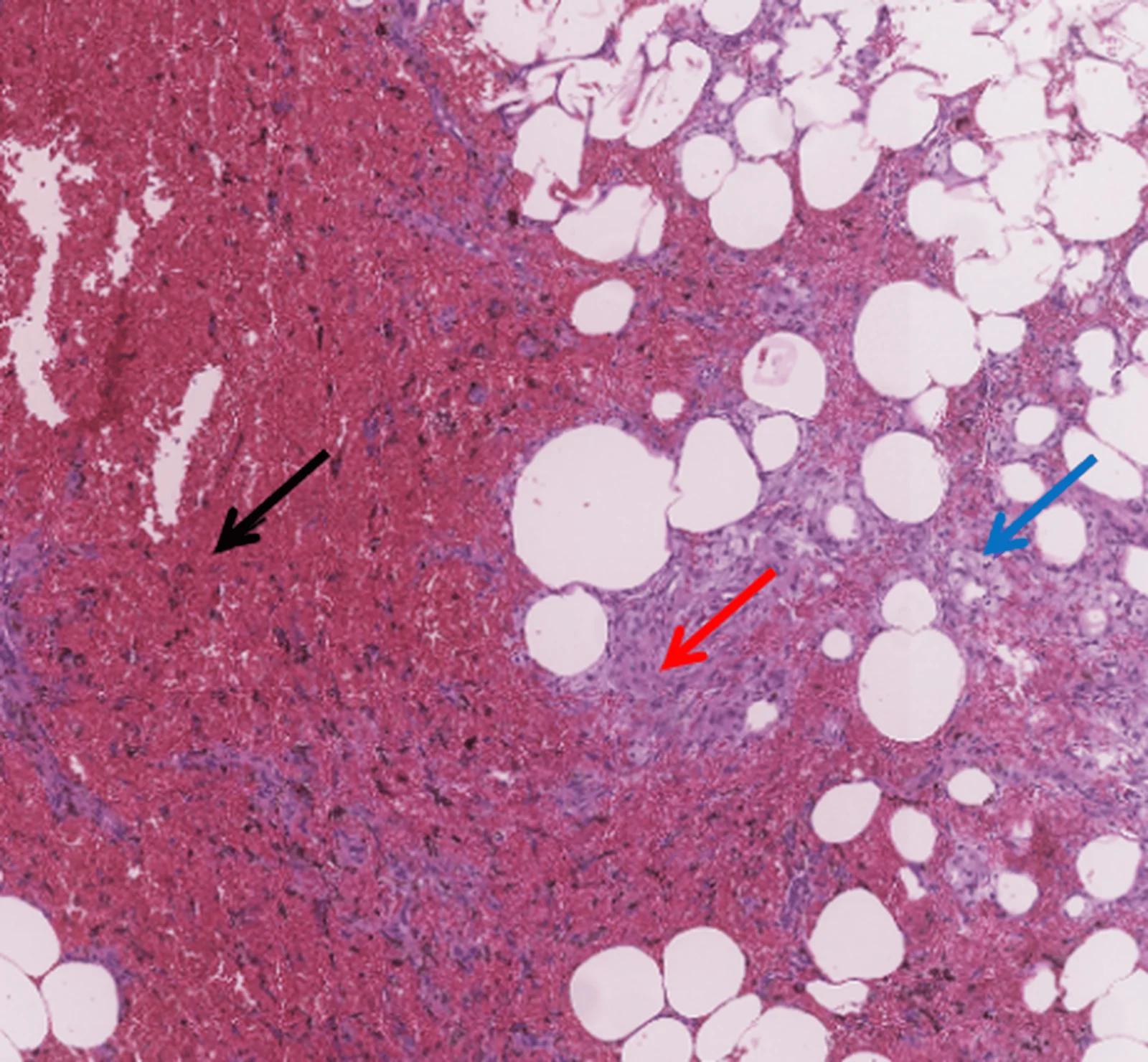
Histopathological findings of a lipedema nodule Histologic image (100×; 10× ocular and 10× objective lenses; hematoxylin-eosin stain) of Lipedema Dermal and Hypodermal Classification 3 revealing adipocytes surrounded by an area of hemorrhage and hemosiderin deposition (black arrow), fibrous proliferation (red arrow), and a focus of steatonecrosis (blue arrow).

Histopathologic evaluation demonstrated adipocytes surrounded by hemorrhage and hemosiderin deposition, together with fibrous proliferation and focal steatonecrosis (Figure 3).

Case 2

A 36-year-old female patient had a clinical diagnosis of lipedema involving the thighs, legs, and arms, with a five-year history of lipedema-related symptoms. She presented with one painful LDHC 3 nodule on the posterior aspect of the right leg and three additional nodules on the posterior aspect of the left thigh as incidental findings. Ultrasound assessment showed the LDHC 3 pattern, including disorganization of the superficial hypodermis, irregular septa, bulging of the deep hypodermis, and irregularity of the DHJ. The nodules were hyperechoic and painful to palpation, with absent or minimal Doppler flow. Histopathologic analysis of the biopsied nodules showed hemorrhage, hemosiderin deposition, fibrous proliferation, and focal steatonecrosis.

Case 3

**Figure 4:**
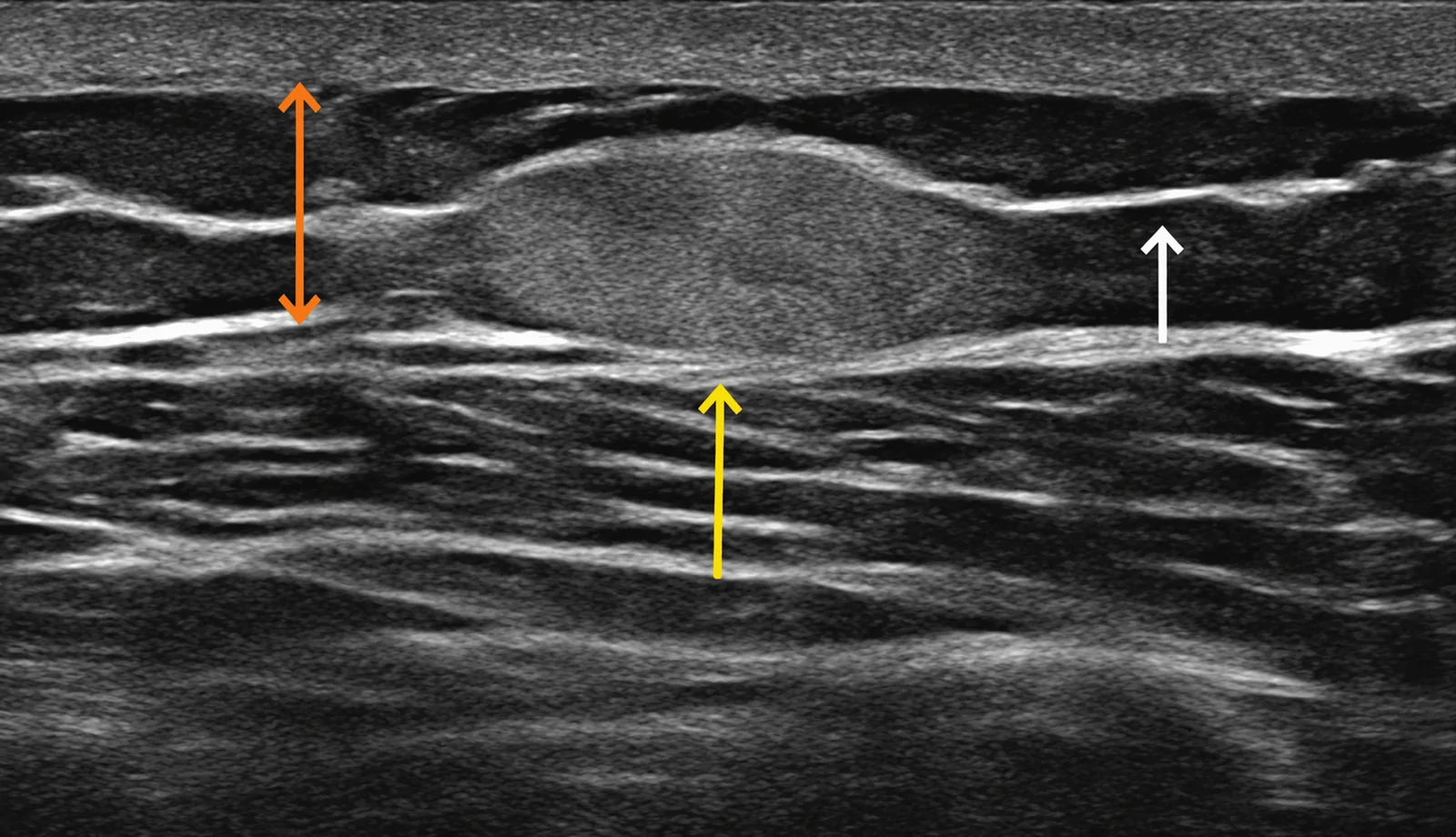
Ultrasound of a thigh angiolipoma Ultrasonographic image of the thigh in a patient with angiolipoma demonstrating normal hypodermis thickness (orange arrow) and an oval hyperechoic nodule (yellow arrow) with regular margins, permeated by adipose tissue with preserved architecture (intact linear echogenic septa, white arrow) and a regular dermal-hypodermal junction.

A 38-year-old female patient presented with a painful subcutaneous nodule on the anterior aspect of the left thigh, with a five-year history of local symptoms. Clinical evaluation ruled out lipedema. Ultrasound showed an oval hyperechoic nodule with regular margins, preserved surrounding adipose architecture, intact linear echogenic septa, a regular DHJ, and absent or minimal Doppler flow (Figure 4).

**Figure 5:**
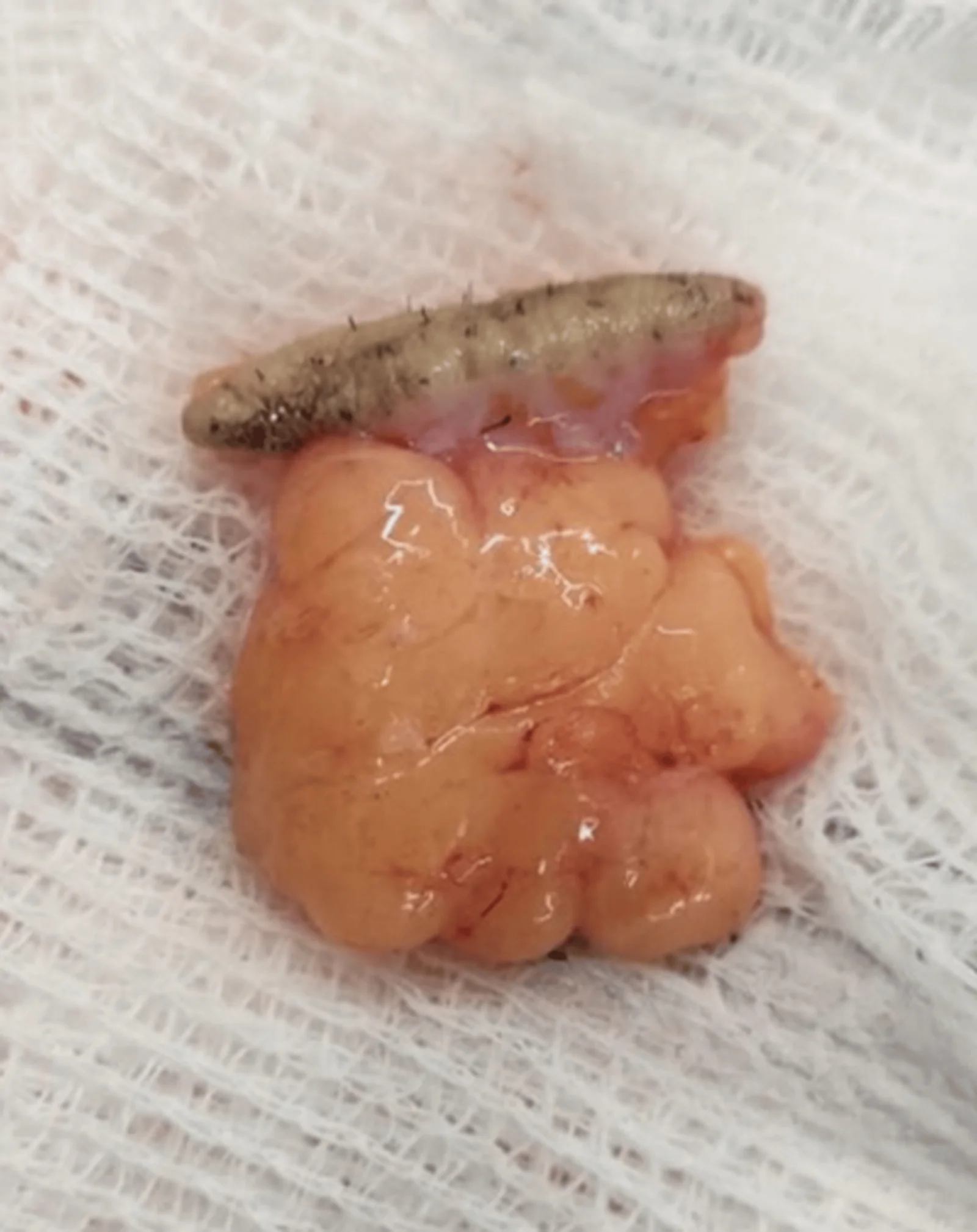
Macroscopic surgical specimen of an angiolipoma Macroscopic image of the angiolipoma surgical specimen demonstrating a well-defined oval lesion with homogeneous yellow-orange coloration due to its lipomatous content.

Macroscopic analysis revealed a well-defined oval nodular lesion with homogeneous yellow-orange coloration due to its lipomatous content (Figure 5).

**Figure 6:**
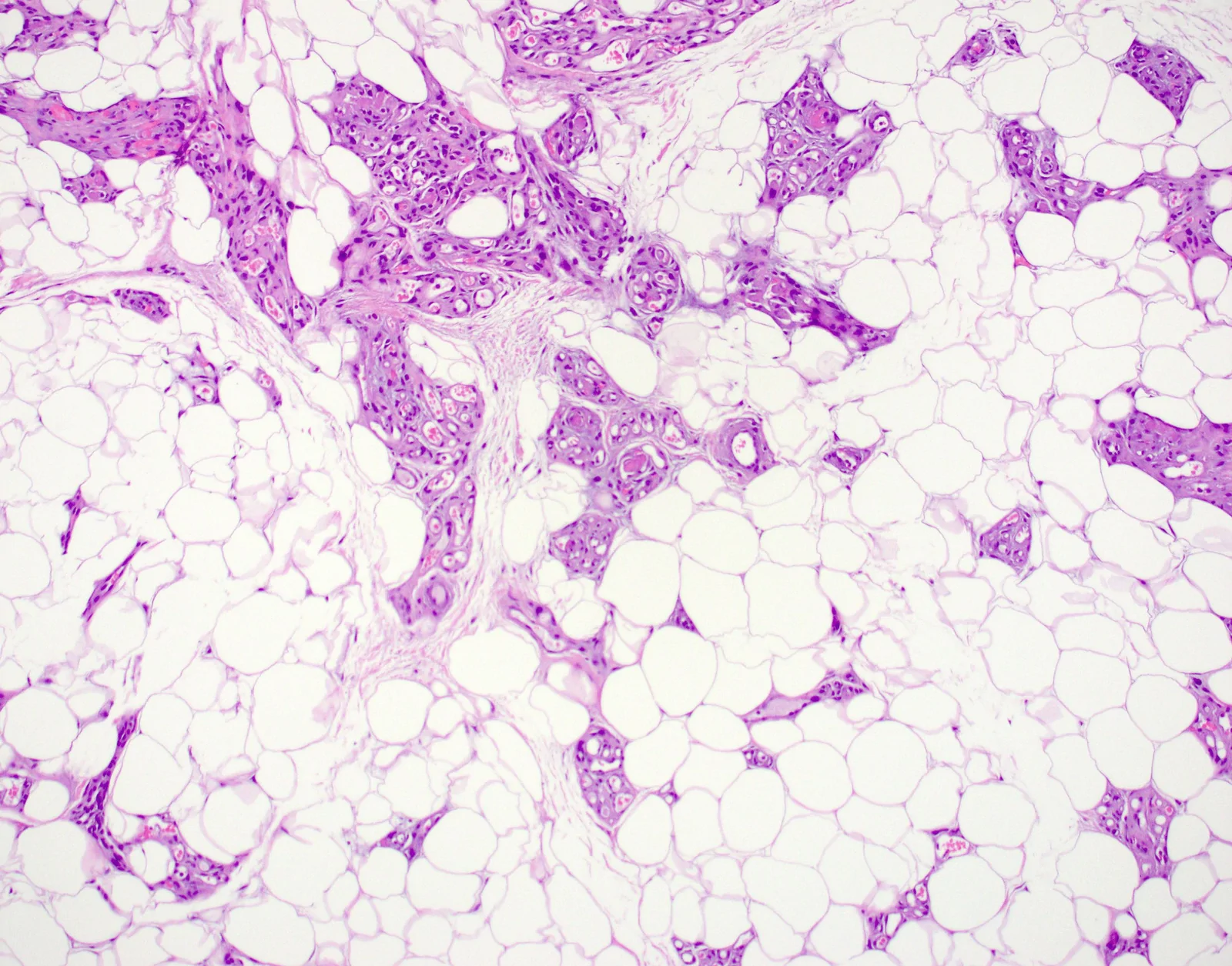
Histopathological findings of an angiolipoma Histologic image (200×; 10× ocular and 20× objective lenses; hematoxylin-eosin stain) of angiolipoma revealing an accumulation of mature, well-differentiated adipocytes of similar size, surrounded by connective tissue. No hemorrhage, cellular atypia, necrosis, or mitoses were observed.

Histologic evaluation demonstrated an accumulation of mature, well-differentiated adipocytes of similar size, surrounded by connective tissue, without hemorrhage, cellular atypia, necrosis, or mitotic activity. The final diagnosis was angiolipoma (Figure 6).

Case 4

A 32-year-old female patient presented with a painful subcutaneous nodule on the anterior aspect of the right thigh, with a two-year history of local symptoms. Clinical diagnosis of lipedema was ruled out. Ultrasound showed an oval hyperechoic nodule with regular margins, preserved surrounding adipose architecture, regular DHJ, and absent or minimal Doppler flow. Histopathologic evaluation confirmed angiolipoma, showing mature adipocytes associated with a connective tissue component and no evidence of hemorrhage, cellular atypia, necrosis, or mitotic activity.

## Discussion

This case series showed that painful hyperechoic nodules in lipedema have a distinct US-pathology correlation and should not be interpreted simply as angiolipomas. In the present cases, the combination of sonographic background alterations and histopathologic findings supports the interpretation that these nodules represent focal chronic hemorrhagic-inflammatory changes occurring within an abnormal hypodermal environment rather than a true adipocytic neoplasm [8-10].

Lipedema is increasingly recognized as a disorder of adipose tissue hypertrophy and hyperplasia associated with chronic inflammation, fibrosis, tissue hypoxia, and vascular fragility [2]. Within this altered microenvironment, immature and fragile capillaries may predispose to microhemorrhage, hemosiderin deposition, and steatonecrosis, findings that correspond histologically to the painful hyperechoic nodules identified on US [8-10]. These observations agree with previous reports from the same research group, which described similar correlations between LDHC 3 nodules and hemorrhagic foci in lipedema [8-10].

Angiolipomas constitute the most relevant tumor-related differential diagnosis because they may be painful and may also appear as hyperechoic nodules on US [11,13,14]. Histologically, they are characterized by mature adipocytes associated with a prominent vascular component, often with fibrin microthrombi [11,12]. However, in the present series, the angiolipoma cases preserved the surrounding US adipose architecture, unlike the lipedema nodules, which were consistently observed within a disorganized superficial hypodermal background. This contextual difference appears to be one of the most useful elements for differential diagnosis.

The LDHC is a qualitative US classification of dermal and hypodermal remodeling that evaluates septa, hyperechoic nodules, dermal thickening, and the DHJ, stratifying findings into four patterns of increasing structural disorganization [7,8]. Within this framework, LDHC 3 is characterized by bulging of the deep hypodermis, disorganization of the superficial hypodermis, irregular septa, hyperechoic nodules, asymmetric dermal thickening, and DHJ irregularity [7,8]. In this sense, the present results reinforce that the sonographic interpretation of these lesions should not be limited to the nodule itself.

From a practical sonographic standpoint, the surrounding hypodermal architecture appears to be the most valuable distinguishing feature. In lipedema, the examiner should not evaluate the nodule in isolation, but rather in association with adjacent findings such as irregular septa, superficial hypodermal disorganization, bulging of the deep hypodermis, and DHJ irregularity [7,8]. This broader tissue context may act as a form of virtual biopsy, helping the radiologist or sonographer suspect a reactive chronic hemorrhagic-inflammatory lesion rather than an angiolipoma [8-10]. The main distinguishing clinical, sonographic, and histopathologic features of LDHC 3 nodules and angiolipomas are summarized in Table 1.

**Table 1:** Clinical, ultrasonographic, and histopathological features aiding in the differentiation between angiolipoma and Lipedema Dermal and Hypodermal Classification 3 nodules Data summarized from references [7-14]. Abbreviations: DHJ, dermal-hypodermal junction; LDHC, Lipedema Dermal and Hypodermal Classification.

Characteristic	Angiolipoma	Hyperechoic Nodule in Lipedema (LDHC 3)
Etiology	Benign adipocytic neoplasm	Focal hemorrhage
Pathophysiology	Uncertain	Hypoxic-ischemic alterations in inflammatory-fibrotic tissue, neovascularization, and capillary fragility
Location	Trunk and extremities	Areas affected by lipedema (thighs, arms, and legs)
Pain	Painful on palpation	Focal and intense pain
Ultrasound	Hyperechoic oval or round-shaped nodule with well-defined borders	Hyperechoic focus with variable margins; may show subtle posterior acoustic shadowing or an internal anechoic area, depending on the stage of hemorrhage. Irregular DHJ, bulging deep hypodermis, and irregular septa with rupture predominantly in the superficial hypodermis
Capsule on ultrasound	Rare	Absent
Vascularization (Doppler)	Frequently absent or with a few vessels	Absent or minimal peripheral flow
Histology	Mature adipocytes with capillary proliferation containing fibrin microthrombi	Hemorrhage with hemosiderin deposition; steatonecrosis; inflammatory infiltrate (eosinophilic granular content and histiocytic reaction)
Hemorrhage	Absent	Present
Hemosiderin	Absent	Present

This study has limitations, including its small sample size, observational design, absence of extensive statistical analysis, limited number of biopsied lesions, and reliance on qualitative image interpretation. Larger multicenter studies are needed to validate these criteria, assess interobserver reproducibility, and define diagnostic performance more precisely. Even so, the present series suggests that hyperechoic nodules in lipedema are distinct from angiolipomas in both pathophysiology and imaging context. Recognizing this distinction may improve diagnostic confidence and help avoid unnecessary invasive procedures.

## Conclusions

Painful hyperechoic nodules in lipedema differ from angiolipomas because they occur within a remodeled hypodermal background and correspond histologically to chronic hemorrhagic-inflammatory change rather than adipocytic neoplasia. US may be a key tool for this distinction, especially when the nodule is interpreted together with surrounding findings such as irregular septa, bulging of the deep hypodermis, and DHJ irregularity. Integrating imaging with clinical evaluation and, when available, histopathology may improve diagnostic accuracy and help avoid unnecessary invasive procedures.

Regarding the outcomes of the presented cases, surgical excision was performed for all nodules due to the initial clinical suspicion of angiolipoma. However, based on the findings of this study, while true angiolipomas are typically treated with surgical excision, hyperechoic nodules in lipedema can be managed conservatively. For these lipedema-associated nodules, serial ultrasound follow-up is recommended to monitor reabsorption of the hemorrhagic focus, thereby preventing inappropriate treatments and unnecessary invasive procedures in future cases.
